# Transcriptome Analysis on the Quality of *Epimedium koreanum* in Different Soil Moisture Conditions at Harvesting Stage

**DOI:** 10.3390/genes15050528

**Published:** 2024-04-23

**Authors:** Yonggang Zhang, Dantong Wang, Feng Wu, Xiangdi Huang, Xiaowei Chai, Limin Yang

**Affiliations:** Cultivation Base of State Key Laboratory for Ecological Restoration and Ecosystem Management of Jilin Province and Ministry of Science and Technology, College of Chinese Medicinal Materials, Jilin Agricultural University, Changchun 130118, China; zhangyonggang@jlau.edu.cn (Y.Z.); DantongWang2024@outlook.com (D.W.); 15270190358@163.com (F.W.); 18093401236@163.com (X.H.); 15754353940@163.com (X.C.)

**Keywords:** *Epimedium koreanum* Nakai (*E. koreanum*), flavonoids, transcriptomics, flavonoid biosynthesis

## Abstract

*Epimedium koreanum* is a traditional Chinese tonic herb. Its main medicinal components are secondary metabolites such as flavonoids and flavonol glycosides, but the biosynthetic mechanism is still unclear. Moisture conditions are a key environmental factor affecting *E. koreanum* medicinal components during harvesting. Different stages of *E. koreanum* under natural conditions after rainfall were selected to study changes in physiological properties, herb quality, and transcriptome. Malondialdehyde (MDA) content increased significantly in the D3 stage after rainfall, and protective enzyme levels also rose. Additionally, the flavonol glycoside content was relatively high. We sequenced the transcriptomes of D1, D3, and D9 (R) and identified differentially expressed genes (DEGs) related to flavonoid synthesis. This analysis allowed us to predict the roadmap and key genes involved in flavonoid biosynthesis for *E. koreanum*. These results suggest that the *E. koreanum* quality can be enhanced by natural drought conditions in the soil after precipitation during harvest. The harvesting period of *E. koreanum* is optimal when soil moisture naturally dries to a relative water content of 26% after precipitation. These conditions help *E. koreanum* tolerate a certain level of water scarcity, resulting in increased expression of flavonoid-related genes and ultimately enhancing the quality of the herb.

## 1. Introduction

*E. koreanum* Nakai is a perennial herb from the Berberidaceae family [1], mainly found in the Changbai Mountain area of Northeastern China, North Korea, and the coast of the Sea of Japan [2,3]. It typically grows in wooded areas, bushes, or semi-shady environments at the edge of forests [4]. The dried leaves of *E. koreanum* are used in traditional Chinese medicine as a medicinal herb known for their ability to tonify the kidneys and Yang, strengthen muscles and bones, and alleviate rheumatism [5]. This herb has been used for thousands of years as a tonic, aphrodisiac, and antirheumatic drug [6]. Modern studies have shown that *E. koreanum* and its fractions exhibit a wide range of pharmacological and biological activities, including antidiabetic [7], anti-fibrosis, antiviral, antioxidant, anti-tumor, antithrombotic, and anti-atherosclerotic properties [8,9]. Studies have shown that the primary bioactive components responsible for the diverse biological activities of the Epimedium plant are isoprenylated flavonol glycosides [10]. These compounds are the final products of the flavonol branch of the flavonoid biosynthesis pathway and are predominant components of *E. koreanum*. Among them, icariin, along with epimedin A, B, and C, has been used as a representative biomarker for quality assessment and chemical taxonomy. However, the mechanism of biosynthesis and regulation of these flavonoid-derived active constituents in *E. koreanum* is unclear. The genes involved in flavonoid synthesis are also unknown.

Water conditions are crucial abiotic environmental factors that influence normal plant growth. Malondialdehyde (MDA) is the ultimate decomposition product of membrane lipid peroxidation, which occurs when plants are affected by drought. After being released from the production site on the membrane, MDA can react with proteins and nucleic acids, disabling them, relaxing the bridge bonds between cellulose molecules, or inhibiting protein synthesis. Therefore, MDA accumulation may damage the membrane and cells. It can also reflect the extent of oxidative stress damage in plant cells [11] and indirectly indicate the level of stress damage in plants.

In response to water deficit conditions, plants react and adapt to stress through intricate molecular responses, such as enhancing protective enzyme activities and synthesizing osmoregulatory substances. Plants respond to drought by increasing antioxidant enzyme activity, which removes reactive oxygen species to alleviate stress caused by drought. In the antioxidant enzyme system, superoxide radicals can be made disproportionate to H_2_O_2_ and O_2_ by SOD (superoxide dismutase), while H_2_O_2_ is broken down by POD (peroxidase) and CAT (catalase) [12]. In addition to enzymatic antioxidants (e.g., antioxidant enzymes such as SOD and POD), non-enzymatic antioxidants (e.g., flavonoids) are also involved in antioxidant defense in plants. Flavonoids and other non-enzymatic antioxidants act as reactive oxygen species (ROS) scavengers by localizing and neutralizing free radicals before they damage cells [13]. Under drought stress, the flavonoid biosynthetic pathway is activated, leading to the accumulation of various flavonoids with the potential to remove harmful ROS [14,15]. In response to drought stress, rice activates several genes involved in the flavonoid pathway. Its corresponding reaction to drought stress is associated with the expression of genes in the flavonoid pathway [16]. Numerous studies have shown that certain drought levels induce medicinal plants to produce more secondary metabolites. For example, under short-term drought stress, *Platycodon grandiflorum* synthesizes and accumulates more platycodonin [17]. A certain degree of water stress significantly increases salvinorin B content, and drought promotes the synthesis and accumulation of baicalin in *Salvia miltiorrhiza* Bunge [18]. Therefore, studying the impact of drought on the synthesis of secondary metabolites in *E. koreanum* is crucial for enhancing the herb’s quality.

## 2. Materials and Methods

### 2.1. Plant Materials

The experiment was carried out from May to September 2022 in the Baicao Garden of Jilin Agricultural University. The geographic location was 43°25′46″ N latitude and 125°49′12″ E longitude at an altitude of 251 m. The test material was *E. koreanum* Nakai.

### 2.2. Sample Treatment

After three months of regular field management, irrigation was halted. Previous studies have shown that September is the best time to harvest *E. koreanum*. Soil moisture naturally decreased after the first rainfall in September and increased after another rainfall (D8). In this study, we used *E. koreanum* green leaf stage leaves as experimental materials. We collected 30 leaves as samples from five stages: D1, D3, D5, D7, and D9 (R), with three replicates at each stage. The leaves were collected using a random sampling method after the rainfall. One portion of the samples was dried in an oven at 60 °C for 24 h, while the other was frozen with liquid nitrogen and stored in a refrigerator at −80 °C for later use. Soil moisture indicators were measured and recorded using a soil moisture meter. The average relative soil moisture content was 34.5%, 26.0%, 23.2%, 17.0%, and 36.3% at D1, D3, D5, D7, and D9 (R), respectively.

### 2.3. Instruments

Plant Total RNA Isolation Kit Plus, RE-05021, Chengdu Fukuji Biotechnology Company Limited, Chengdu, China; NovoScript^®^ 1st Strand cDNA Synthesis Kit, E041-01A, Shanghai Nearshore Protein Technology Co., Shanghai, China; NovoScript^®^ SYBR One-Step qRT-PCR Kit, E092-01A, Shanghai Nearshore Protein Technology Co. Shanghai, China; NanoPhotometerTM P-Class Microspectrophotometer, P330, Implen, Germany; CFX96 Real-Time Fluorescence Quantitative PCR (qRT-PCR) Instrument, Bio-Rad, Agilent 1260 Liquid Chromatograph, Agilent Technologies, Inc., Palo Alto, CA, USA.

### 2.4. Experimental Methods

#### 2.4.1. Determination of Epimedin A, Epimedin B, Epimedin C, and Icariin Content

The chromatographic column used was Eclipse XDB-C18 (4.6 mm × 250 mm, 5 µm). The mobile phase consisted of acetonitrile (A) and water (C), the flow rate was 1.0 mL/min, and the column temperature was maintained at 25 °C. Detection was performed at a wavelength of 270 nm and the injection volume was 10 µL (Table 1). Epimedoside content in standard epimedin A, epimedin B, and epimedin C products was 98% or more (purchased from the China Institute for the Control of Pharmaceutical and Biological Products) (Table 2). The standards were purchased from the China Institute for the Control of Pharmaceutical and Biological Products (ICCBP).

#### 2.4.2. Determination of MDA and Antioxidant Enzyme Levels

Plant malondialdehyde (MDA) content was measured using the colorimetric method (A003-3-1). Catalase (CAT) activity was determined through the visible light method (A007-1-1) and peroxidase (POD) activity using kit A084-3-1. Total superoxide dismutase (SOD) concentrations were assessed with the hydroxylamine method (A001-1-2). These measurements were conducted according to instructions provided in kits sourced from Nanjing Jiancheng Bioengineering Institute in Nanjing, China.

#### 2.4.3. Total RNA Extraction and Transcriptome Sequencing

*E. koreanum* leaves were powdered in liquid nitrogen, and total RNA was extracted using the TRK1001 total RNA purification kit (LC Science, Houston, TX, USA). The amount and purity of total RNA were analyzed using a Bioanalyzer 2100 and RNA 1000 Nano LabChip Kit (Agilent, Santa Clara, CA, USA). Poly(A) RNA was purified from total RNA (5 μg) using poly-T oligo-attached magnetic beads. The purification process was divided into two rounds. After purification, the mRNA was fragmented into small pieces using divalent cations at a high temperature. The cleaved RNA fragments were then reverse transcribed following the protocol of the mRNASeq Sample Preparation Kit (Illumina, San Diego, CA, USA) to generate the final cDNA library, which was sequenced using Illumina Novaseq™ 6000 (LC Sciences, Houston, TX, USA). The library construction and sequencing were entrusted to Hangzhou Lianchuan Biotechnology Co., Hangzhou, China.

#### 2.4.4. Differentially Expressed Genes, Enrichment, and Interaction Analysis

Unigene expression was calculated using transcripts per million (TPM) and screened for differentially expressed genes in *E. koreanum* leaves with false discovery rate (FDR) ≤ 0.05 and fold change (FC) ≥ 1 as the screening criteria. Gene ontology (GO) and Kyoto Encyclopedia of Genes and Genomes (KEGG) enrichment analyses were conducted using the clusterProfiler R package (version 3.4.4). A corrected *p*-value of ≤0.05 was selected as the threshold condition for significant enrichment of differential genes in GO terms. KEGG-enriched differential genes were used as reference data for protein interaction analysis of their CDS using String (Nanjing, China).

#### 2.4.5. qRT-PCR Validation

Total RNA from *E. koreanum* leaves at different time points was reverse transcribed into cDNA using the SparkJade^®^ SPARKscript All-in-one RT SuperMix for qPCR (with gDNA Eraser) kit. The template was diluted threefold and used in the subsequent qRT-PCR reaction. The five unigenes and internal reference 18S related to secondary metabolite biosynthesis in the KEGG pathway were selected for qRT-PCR. Specific primers were designed using the predicted CDS sequences and Primer3 Plus software (Table 3). The reaction procedure was based on the SparkJade^®^ 2 × SYBR Green qPCR Mix (With ROX) kit. Relative gene expression was calculated using the 2^−ΔΔCt^ method.

## 3. Results

### 3.1. Content of Active Ingredients

**Figure 1:**
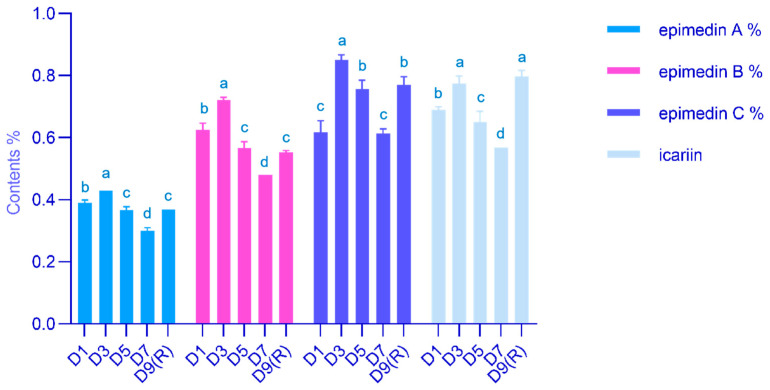
Content of each flavonol glycoside. Different lowercase letters are significant differences at the 5% significance level for different stages of the same content of active ingredientst.

This picture shows the main medicinal components at various time points after harvesting *E. koreanum* (Figure 1). Epimedin A, epimedin B, epimedin C, and icariin levels increased significantly by 10.26%, 14.89%, 37.84%, and 12.08%, respectively, at the D3 stage compared to the D1 stage. The content of each flavonoid glycoside decreased significantly with prolonged precipitation and increased after another precipitation. Specifically, icariin increased by 15.46% at D9 (R) compared to D1.

### 3.2. MDA and Protective Enzyme Content

**Figure 2:**
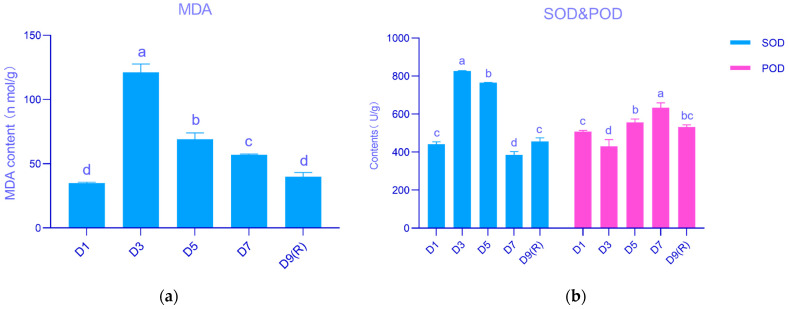
Research on MDA content and protective enzyme activities in various stages following rainfall. (**a**) MDA content, (**b**) SOD and POD contents. Different lowercase letters are significant differences at the 5% significance level for different stages of the same protective enzyme.

Figure 2a shows the effects of different stages after rainfall on *E. koreanum* MDA content at the harvesting stage. MDA content increased significantly at D3, reaching 121.01 nmol/g, then decreased significantly. This decrease can be attributed to the positive regulation of the protective enzyme system, antioxidant substances, and other beneficial factors, leading to a gradual decline in MDA content. Changes in soil moisture were analyzed for their effect on the protective enzyme activities of *E. koreanum* at the harvesting stage (Figure 2b). The superoxide dismutase (SOD) content was significantly higher than other groups during the D3 stage, while the peroxidase (POD) content began to increase gradually during the same stage. The results showed that moisture changes after precipitation affected SOD and POD activities in *E. koreanum*, but the extent of their effects varied.

### 3.3. Sequencing Quality of E. koreanum RNA

**Figure 3:**
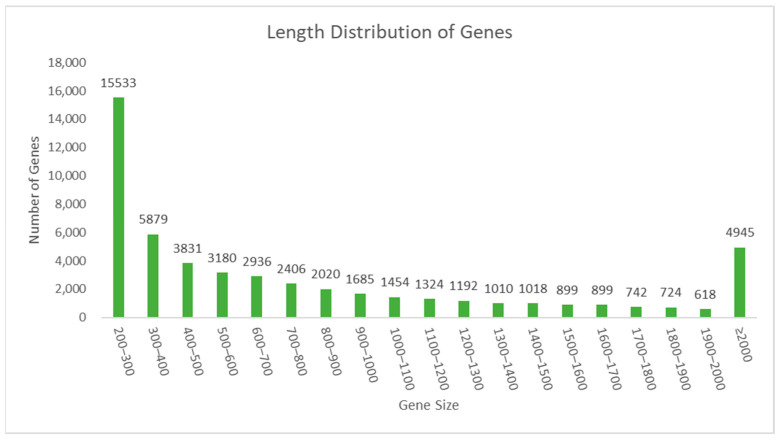
The lengths of the genes.

**Figure 4:**
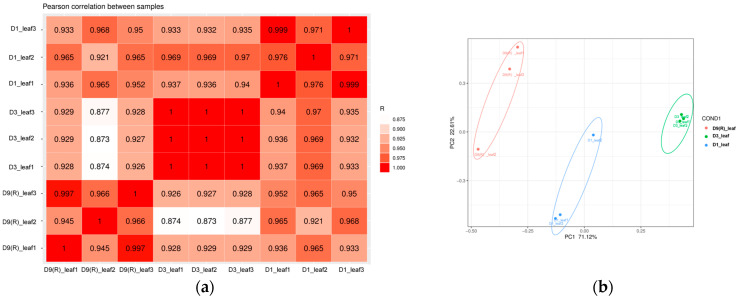
Correlation analysis and principal component analysis (PCA): (**a**) sample correlation analysis; (**b**) sample distributions in PCA 1 and PCA 2.

A total of nine transcriptome libraries were obtained, with the number of raw reads ranging from 39,017,882 to 49,464,974. The GC content ranged from 44.89% to 45.30%, the base quality value Q20 (sequencing error rate less than 0.01) ranged from 97.99% to 98.22%, and the value of Q30 (sequencing error rate less than 0.001) ranged from 93.61% to 94.19% (Table 4). A total of 52,295 genes were assembled (Figure 3). The R^2^ value of three samples from the same treatment exceeded 0.873 (Figure 4a). According to the principal component analysis (PCA) (Figure 4b), samples from different treatments were separated, and samples from the same treatment were aggregated, indicating a considerable correlation among the repetitions.

### 3.4. Analysis of Differentially Expressed Genes

**Figure 5:**
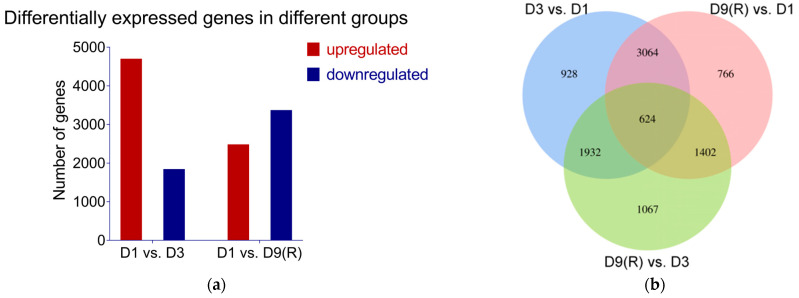
Differentially expressed genes (DEGs): (**a**) numbers of DEGs in D1 vs. D3; D1 vs. D9 (R); (**b**) Venn diagrams for D1 vs. D3; D9 (R) vs. D1 and D9 (R) vs. D1.

A total of 9783 differential genes were identified in this study. There were significantly more upregulated genes than downregulated genes in D1 vs. D3, and significantly more downregulated genes than upregulated genes in D1 vs. D9 (R) (Figure 5a). A total of 624 differentially expressed genes (DEGs) were identified as coexpressed in two-by-two Wayne plots across the three stages (Figure 5b). The above results indicate that these genes responded differently to changes in soil moisture, and a total of 624 genes played a role after precipitation.

### 3.5. GO Enrichment Analysis

**Figure 6:**
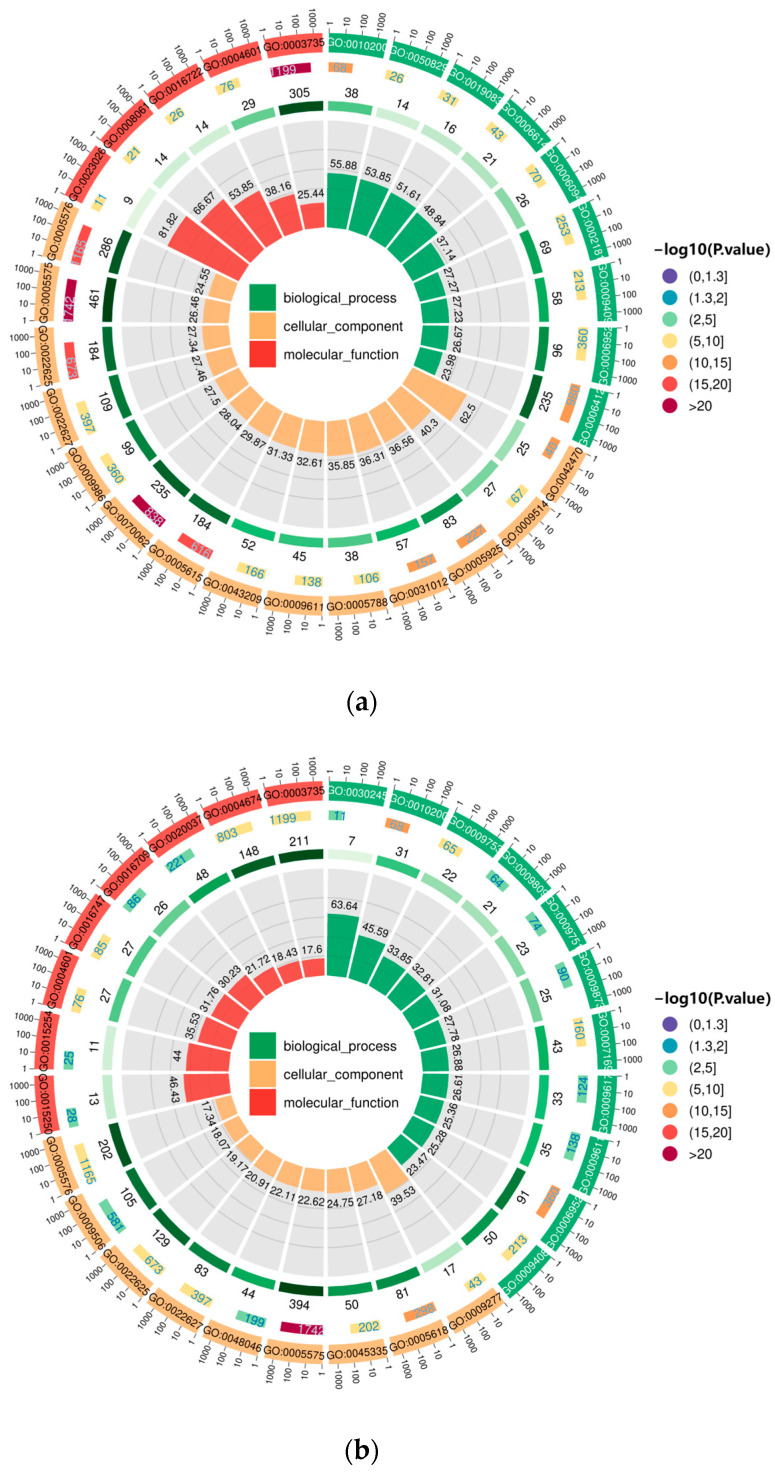

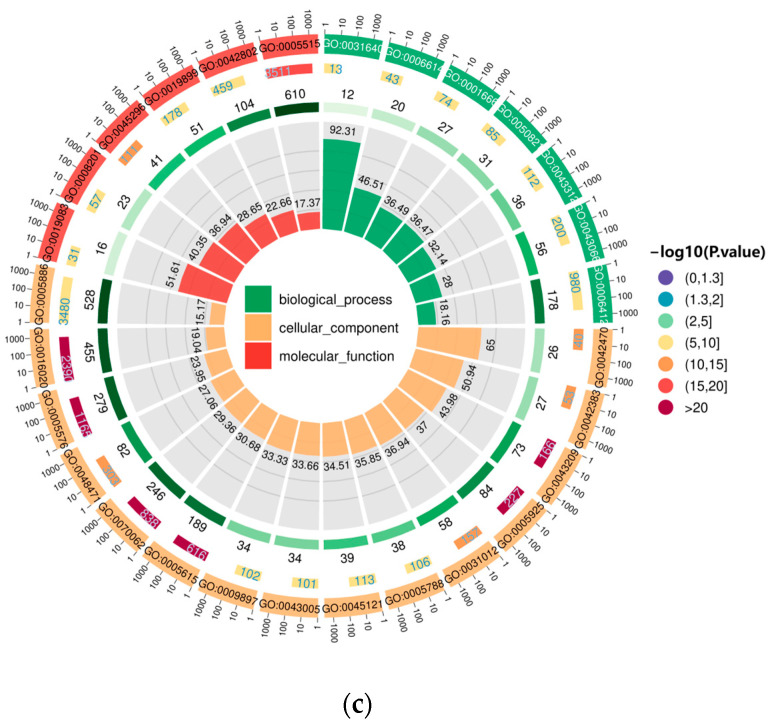
Gene Ontology (GO) enrichment analysis in the following groups: (**a**) D1 vs. D3; (**b**) D9 (R) vs. D1; (**c**) D9 (R) vs. D1.

For D1 vs. D3, DEGs for D9(R) vs. D1 and D9(R) vs. D1 were annotated in Gene Ontology (GO) (Figure 6). Selected according to *p* < 0.05, the distribution of DEGs in the leaves of *E. koreanum* was consistent across biological processes, cellular components, and molecular functions. DEGs were primarily associated with metabolic processes, cellular processes, stimulus responses, cell locations, cell membranes, catalytic activities, and other functions.

### 3.6. KEGG Enrichment Analysis

**Figure 7:**
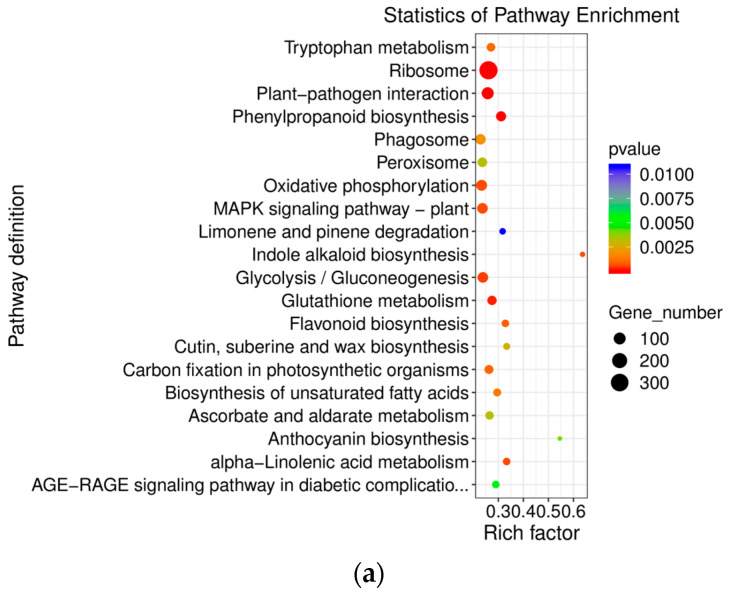

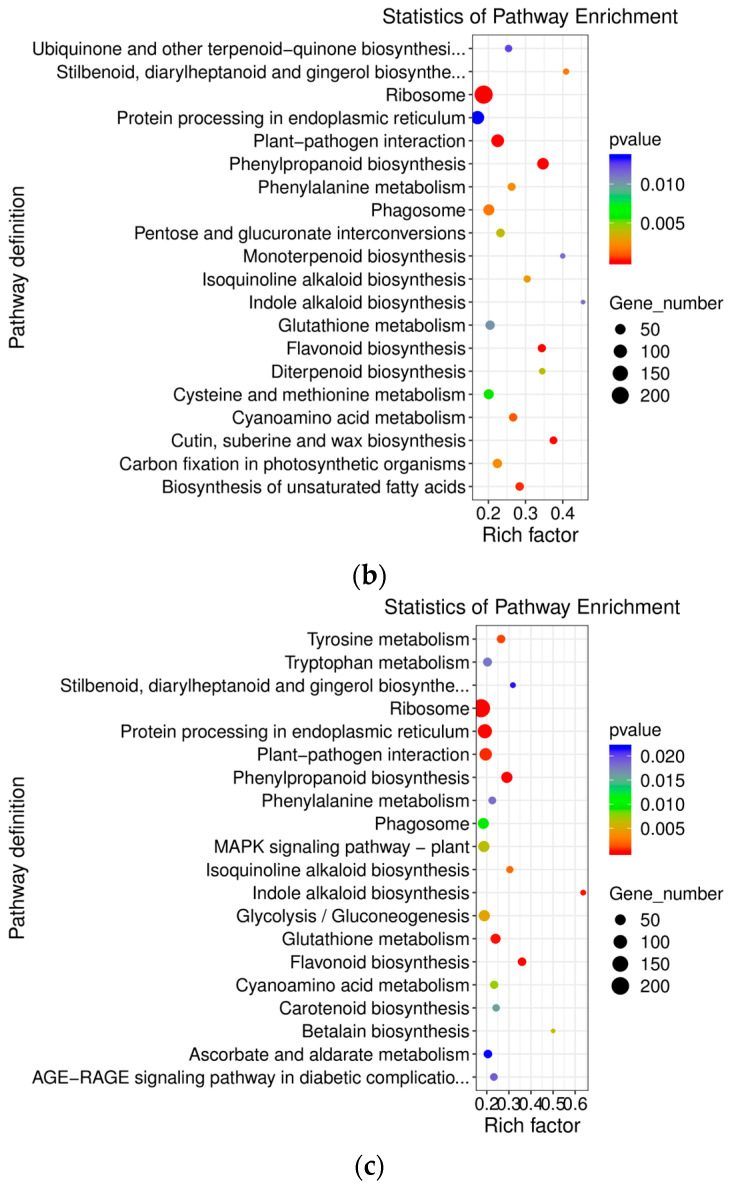
Kyoto Encyclopedia of Genes and Genomes (KEGG) enrichment analysis in the following groups: (**a**) D1 vs. D3; (**b**) D9 (R) vs. D1; (**c**) D9 (R) vs. D3.

According to the screening criterion of *p* ≤ 0.05, a total of 43 KEGG key pathways were significantly enriched in gene expression. Among them, D1 vs. D3, D (R) vs. D3, and D9 (R) vs. D1 had 1429, 970, and 1060 differentially expressed genes enriched in 31, 24, and 28 pathways, respectively. KEGG enrichment pathways mainly included the ribosome, phenylpropane biosynthesis pathway, flavonoid biosynthesis pathway, and MAPK signal transduction pathway (Figure 7).

### 3.7. Gene Expression Analysis and Key Gene Screening of Flavonoid Synthesis Pathway in E. koreanum

**Figure 8:**
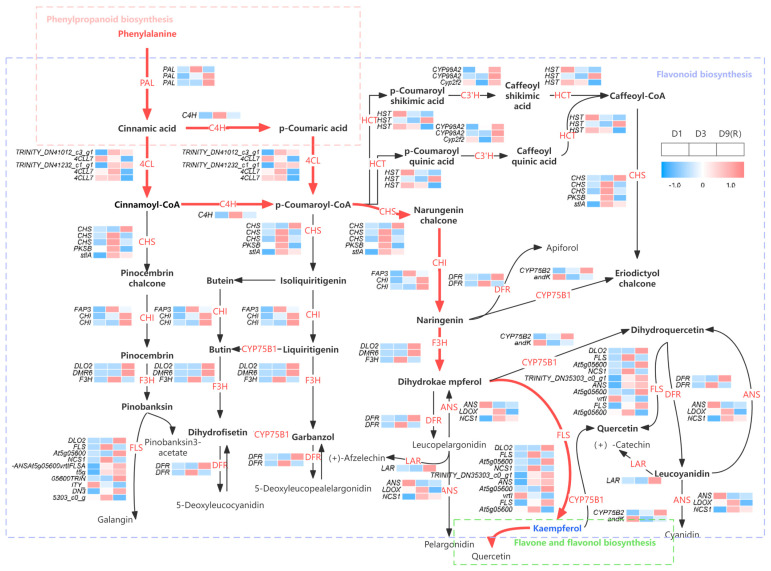
Flavonoid metabolic pathways in *E. koreanum*. The red part may be the key pathway.

A total of 81 genes were identified in the flavonoid metabolic pathway. Among them, a total of 48 genes were either upregulated or downregulated in flavonoid biosynthesis expression, indicating that flavonoids participated in gene regulation in *E. koreanum* after rainfall. The pathways involved in flavonoid biosynthesis in *E. koreanum* were mapped at various times after rainfall during the harvesting period (Figure 8). As shown in Figure 8, most of the genes were highly expressed in D3, followed by some genes with high expression in D9 (R), and basically low expression in D1. These results are essentially in line with modifications in epimedin A, epimedin B, epimedin C, and icariin content, indicating that the D3 stage is more favorable for the expression of flavonoid biosynthesis pathway genes in *E. koreanum*. The main components of *E. koreanum* are isoprenylated flavonol glycosides, which are the end products of the flavonol branch of the flavonoid biosynthetic pathway. The red part may be the key pathway for the pharmacological components of *E. koreanum*, and *PAL*, *C4H*, *CHS*, *CHI*, *F3H,* and *FLS* may be the key genes for synthesizing the pharmacological components of *E. koreanum.*

### 3.8. Correlation Analysis between the Quality of E. koreanum and Influencing Factors

**Figure 9:**
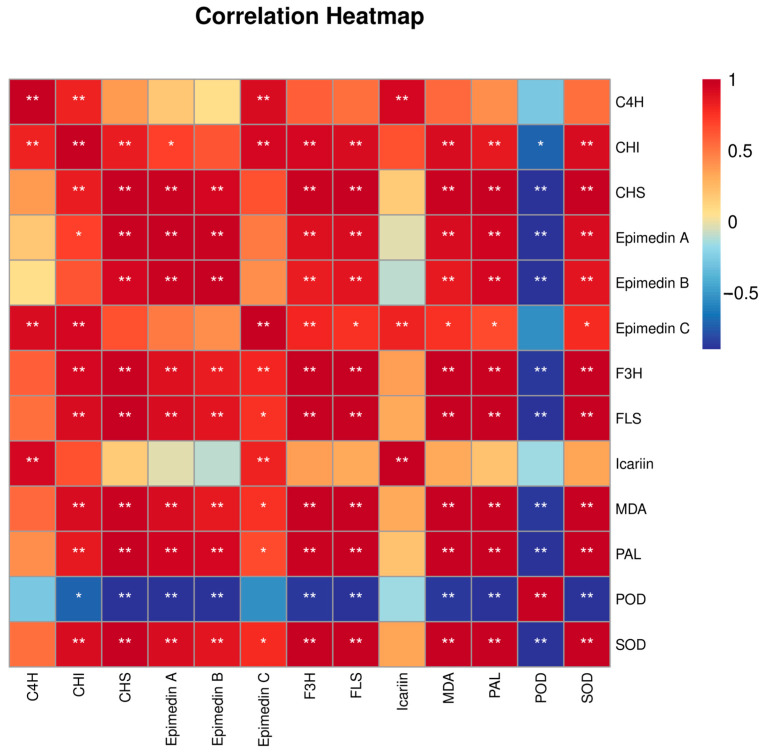
Correlation between quality and influencing factors of E. koreanum.* *p* < 0.05 ** *p* < 0.01.

Correlation analysis of four indicators of *E. koreanum* quality, namely, epimedin A, epimedin B, epimedin C, and icariin, with MDA, protective enzymes, and the six key enzyme genes screened, revealed that epimedin A and epimedin B contents were both significantly positively correlated with SOD and MDA contents and the gene expressions of *PAL*, *FLS*, *F3H,* and *CHS* (Figure 9). Epimedin C content was positively correlated with *F3H*, *C4H,* and *CHI* expressions in a highly significant manner. Icariin content was significantly and positively correlated with *C4H* expression only.

### 3.9. Analysis of Protein Interactions and Prediction of Key Proteins in Flavonoid-Synthesizing Genes in E. koreanum

**Figure 10:**
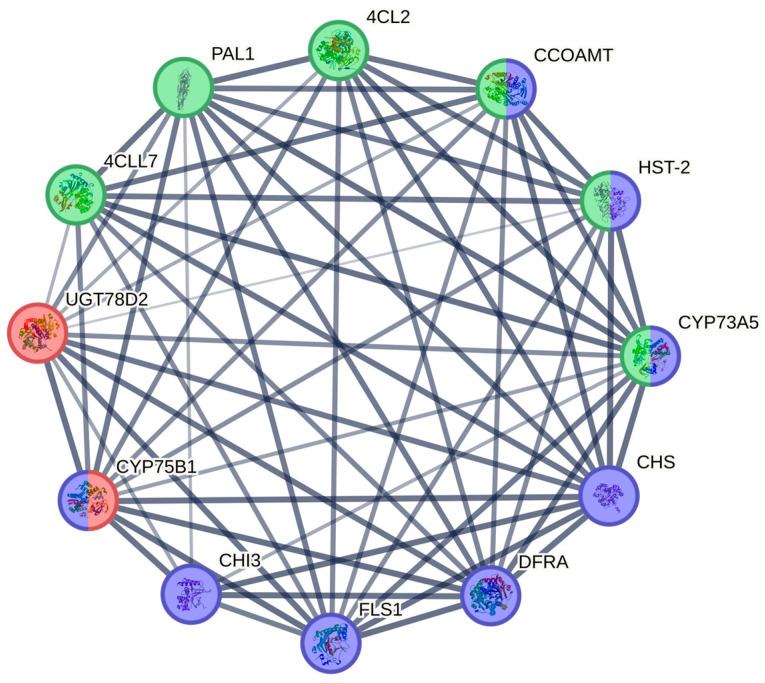
The protein interaction network shows the strength of data support through varying line thickness. The colors red, green, and blue correspond to KEGG annotations for flavonoid, flavonol, and phenylpropane biosynthetic pathways, respectively.

Protein–protein interaction networks were constructed and analyzed for protein sequences involved in phenylpropanoid biosynthesis, flavonoid biosynthesis, and flavone and flavonol biosynthesis. These sequences were identified from differentially expressed genes in three stages of *E. koreanum.* Biosynthesis protein sequences were constructed to analyze the protein–protein interaction network (Figure 10). According to the protein–protein interaction network diagram, PAL, 4CL2, 4CL7, CCOAMT, HST-2, CYP73A5 (C4H), CHI3, CHS, DFRA, LDOX, FLS1, CYP75B1, and UGT78B2 proteins interact with each other. This interaction may explain why the synthesis of epimedin A, epimedin B, epimedin C, and icariin in *E. koreanum* was affected at different stages after rainfall.

### 3.10. qRT-PCR Validation of RNA-seq Data

**Figure 11:**
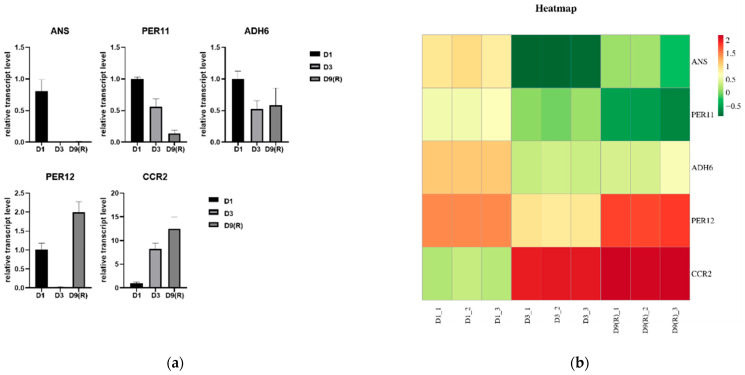
Expression of genes *ANS*, *PER11*, *ADH6*, *PER12* and *CCR2* in quantitative real-time RNA-seq and PCR (qRT-PCR). (**a**) Expression in PCR (qRT-PCR).(**b**) Heatmap of RNA-seq expression TPM values.

To validate the accuracy of RNA-seq data in this study, five genes possibly related to changes in flavonoids at different stages, including phenylpropane biosynthesis and flavonoid biosynthesis-related gene coding pathways, were detected using qRT-PCR (Figure 11a). Correlation analysis showed that the qRT-PCR results were consistent with the trend of RNA-seq expression levels (Figure 11b), indicating the reliability of transcriptome data.

## 4. Discussion

Moisture is one of the most important abiotic environmental factors affecting plant growth and development. Plants have developed a complex gene expression regulatory network to adapt to these conditions [19]. Reference-free transcriptome sequencing technology can offer valuable data resources for the comprehensive analysis of plant gene expression.

In this study, we measured flavonol glycosides, MDA, and protective enzyme contents, conducted transcriptome sequencing, and analyzed differentially expressed genes in the leaf tissues of *E. koreanum* at various stages following precipitation. The results showed that during the D3 stage after rainfall, MDA content increased, the cell membrane was damaged, the protective enzyme system became activated, and medicinal components accumulated rapidly. Therefore, a certain degree of water deficit is more favorable for flavonoid synthesis and accumulation due to the excess reactive oxygen species (ROS) caused by low water content [20], which triggers the synthesis of antioxidant components such as flavonoids to protect the plant from damage [21]. However, there were differences in the synthesis and accumulation of icariin and other flavonoid components. These variances can be attributed to changes in soil moisture levels, leading to modifications in the sugar group structure of *E. koreanum* in varying ways and to different extents. O-glycosyltransferases (O-GTs) are known to contribute to the biosynthesis of various glycosides in licorice [22]. It is also possible that pathogenic microorganisms and fungi vary under different soil moisture conditions. To protect against damage from biotic and abiotic stresses, the plant synthesizes flavonoids that act as antitoxins or antioxidants [23]. Endophytic fungus (Rhizobium rhizolycopersici GUH21) has been shown to significantly enhance the accumulation of isoglycyrrhizin and glycyrrhetinic acid in the root system of *Glycyrrhiza glabra* under increased watering and low temperatures [24].

The results of GO enrichment analysis revealed that the distribution of DEGs in the GO functional categories in the leaves of *E. koreanum* during various time stages following rainfall throughout the harvesting season was consistent. These DEGs were predominantly enriched in metabolic processes, cellular structure, stimulus response, binding, transcriptional regulator activity, and catalytic activity. This pattern was akin to the GO analysis findings for the transcriptome of *Sophora tonkinensis* under moderate drought stress [25].

The results of KEGG enrichment analysis revealed that the main pathways enriched in *E. koreanum* leaves were ribosomal, phenylpropanoid biosynthesis, flavonoid biosynthesis, MAPK signaling, and phytopathogen interaction pathways. On the third day, the key enzyme genes *PAL* and *C4H* in the phenylpropanoid pathway were highly expressed. This increased expression can be linked to limited soil moisture, which resulted in the overproduction of ROS [26,27]. Subsequently, enzyme inactivation and lipid peroxidation occurred. To mitigate these damages, cells produced flavonoids as antioxidants to neutralize excess ROS [28].

The protein–protein interaction (PPI) analysis revealed an interaction network between the flavonoid biosynthesis and phenylpropane biosynthesis pathways, as well as the flavonoids and flavonol biosynthesis pathways. The results revealed that the key proteins responsible for the synthesis of flavonoids in *E. koreanum* had intricate interactions, particularly downstream flavonols, UGT78D2, and CYP75B1. UGT78D2, a flavonol 3-O-glucosyltransferase from the UDP-glycosyltransferase family, facilitated the transfer of glucose from UDP-glucose to quercetin and kaempferol at the 3-OH position. Glycosylation is a prominent modification reaction and is usually the final step in flavonoid biosynthesis, where flavonoids exist as glycosides, making them more stable and soluble and facilitating their transport and accumulation in cells [29]. CYP75B1, a flavonoid 3′-monoglycosylase belonging to the cytochrome P450 family, catalyzes the 3′-hydroxylation of the B ring of flavonoids to the 3′,4′-hydroxylated state, converting naringenin to apigenin and dihydrocamptothecin to dihydroquercetin. Light-intensity studies in *Syringa oblata* Lindl have shown that the CYP75B1 gene upregulates rutin biosynthesis. This gene may play a role in regulating the flavonoid biosynthesis pathway as a crucial target in the overall metabolic process [30].

## 5. Conclusions

After assessing *E. koreanum* quality, MDA, and protective enzymes at various intervals following precipitation, the transcriptomes of D1, D3, and D9 (R) were sequenced to explore the impact of different post-precipitation stages on the quality of *E. koreanum*, as well as its associated genes and metabolic pathways. Generally speaking, the concentration of flavonol glycosides in *E. koreanum* was relatively high during natural drought until the relative water content of the soil decreased to 26%. There were obvious differences in gene expression at different stages. Most flavonoid biosynthesis genes were expressed when the relative soil water content decreased to 26%. These differentially expressed genes were enriched in various metabolic pathways. Among them, phenylpropanoid biosynthesis and flavonoid biosynthesis were highly enriched, with the highest DEG content. By combining the results of previous studies and further analysis, it can be inferred that *E. koreanum* experienced some degree of water stress during harvest due to insufficient soil moisture. *E. koreanum* undergoes a series of physiological adjustments to resist environmental stress. The antioxidant enzyme system and secondary metabolites responded positively to natural drought following precipitation, which decreased soil moisture content by 26%. Flavonoid production was regulated by increasing the content of protective enzymes and the expression of enzyme genes associated with flavonoid biosynthesis pathways to adapt to changing soil moisture conditions. These results lay a foundational understanding for the molecular mechanism of *E. koreanum*’s response to soil moisture. They provide theoretical support for enhancing *E. koreanum* quality through some degree of water deficit.

## Figures and Tables

**Table 1:** Gradient elution table of flavonol glycosides from *E. koreanum* leaves.

Time	Gradient Phase A (%)	Gradient Phase C (%)
0~20	24→26	76→74
20~30	26→30	74→70
30~45	30→45	70→55
45~50	45→60	55→40
50~60	60→24	40→76

**Table 2:** Chemical structure of c, epimedin B, epimedin C, and icariin.

Epimedin A	Epimedin B	Epimedin C	Icariin
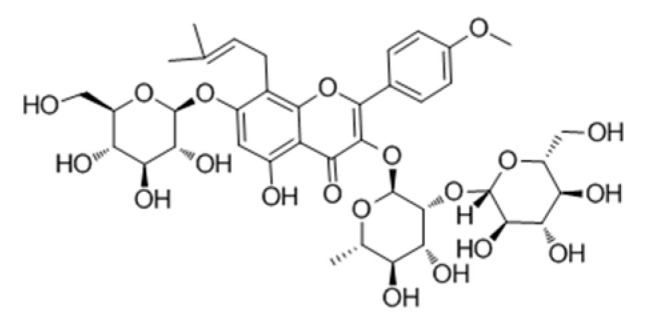	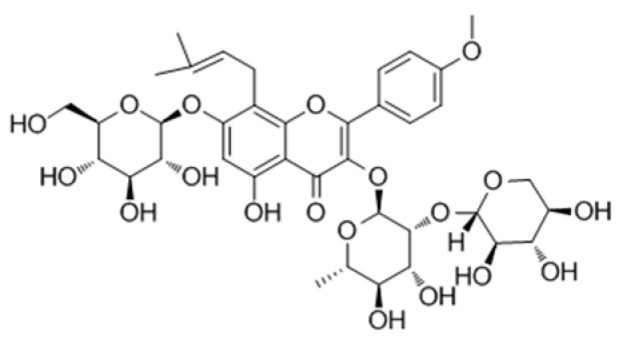	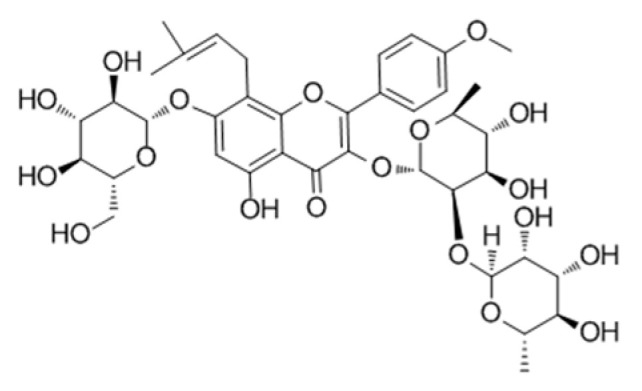	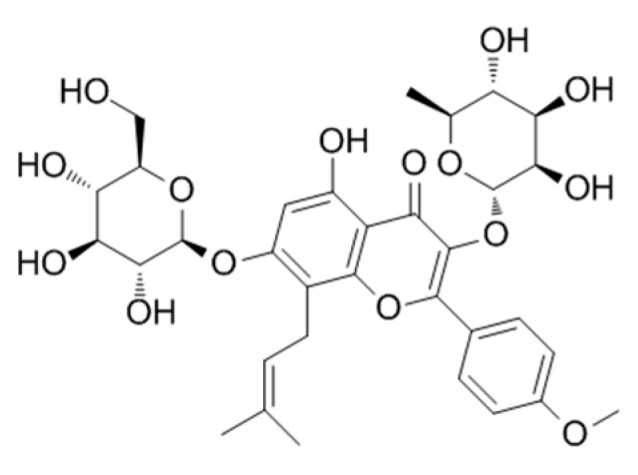

**Table 3:** Specific primers of qRT-PCR amplification.

Name	Forward Primer (5′→3′)	Reverse Primer (5′→3′)
*18S*	CGCTGCGTTCTTCATCGTT	TTGGGTAGTCGGGCTGGTC
*ANS*	ACCAAGGCAGCATTGTTAGC	TGAACAAGGCGGGTAGTAACC
*PER11*	AGCATTGGTTCCCATGCAAC	ACAGCACACTCCATCTCCTTC
*ADH6*	TCACTTCGGCTTGCTATTCG	TCGTCAGTGGCGATGAAATG
*OMT*	ATTCCGTCATCACTGCCTCTAG	ACTTGCAAACAGGTGCCAAG
*CCR2*	TGTCCTAAAGGCATGCGTTG	AAACAGAACAGCGGCAATGG

**Table 4:** Statistics of sequencing results.

Sample	Raw Reads	Raw Bases	Valid Reads	Valid Bases	Valid%	Q20%	GC%
D1-1	43,154,254	6.47 G	42,501,058	6.31 G	98.49	98.06	45.29
D1-2	47,879,254	7.18 G	47,119,460	6.99 G	98.41	97.99	45.03
D1-3	48,121,936	7.22 G	47,358,136	7.02 G	98.41	98.05	45.30
D3-1	40,412,608	6.06 G	39,812,314	5.91 G	98.51	98.18	44.91
D3-2	39,276,616	5.89 G	38,687,458	5.74 G	98.50	98.22	44.91
D3-3	39,017,882	5.85 G	38,438,222	5.71 G	98.51	98.18	44.93
D9(R)-1	45,177,958	6.78 G	44,464,904	6.60 G	98.42	98.1	44.83
D9(R)-2	49,464,974	7.42 G	48,634,656	7.21 G	98.32	98.09	45.12
D9(R)-3	43,015,912	6.45 G	42,379,574	6.29 G	98.52	98.11	44.89

## Data Availability

The datasets used during the current study are available from the corresponding author upon reasonable request due to privacy.
